# Psychosocial impact of undergoing prostate cancer screening for men with *BRCA1 or BRCA2* mutations

**DOI:** 10.1111/bju.14412

**Published:** 2018-06-22

**Authors:** Elizabeth K. Bancroft, Sibel Saya, Elizabeth C. Page, Kathryn Myhill, Sarah Thomas, Jennifer Pope, Anthony Chamberlain, Rachel Hart, Wayne Glover, Jackie Cook, Derek J. Rosario, Brian T. Helfand, Christina Hutten Selkirk, Rosemarie Davidson, Mark Longmuir, Diana M. Eccles, Neus Gadea, Carole Brewer, Julian Barwell, Monica Salinas, Lynn Greenhalgh, Marc Tischkowitz, Alex Henderson, David Gareth Evans, Saundra S. Buys, IMPACT Study Steering Committee, IMPACT Collaborators, Rosalind A. Eeles, Neil K. Aaronson

**Affiliations:** ^1^ Oncogenetics Team Royal Marsden NHS Foundation Trust London UK; ^2^ Oncogenetics Team Institute of Cancer Research London UK; ^3^ Clinical Genetics Unit Birmingham Women's Hospital Birmingham UK; ^4^ Sheffield Clinical Genetics Service Sheffield Children's Hospital Sheffield UK; ^5^ Department of Urology Royal Hallamshire Hospital Sheffield UK; ^6^ John and Carol Walter Center for Urological Health NorthShore University HealthSystem Evanston IL USA; ^7^ Clinical Genetics Department Queen Elizabeth University Hospital Glasgow UK; ^8^ Wessex Clinical Genetics Service Princess Anne Hospital Southampton UK; ^9^ Faculty of Medicine University of Southampton University Hospital Southampton NHS Foundation Trust Southampton UK; ^10^ High Risk and Cancer Prevention Clinic Vall d'Hebron University Hospital Barcelona Spain; ^11^ Clinical Genetics Department Royal Devon and Exeter Hospital Exeter UK; ^12^ Department of Genetics University of Leicester Leicester UK; ^13^ Clinical Genetics University Hospitals Leicester Leicester UK; ^14^ Hereditary Cancer Programme Catalan Institute of Oncology (ICO‐IDIBELL, CIBERONC) L'Hospitalet de Llobregat Barcelona Spain; ^15^ Cheshire and Mersey Clinical Genetics Service Liverpool Women's Hospital Liverpool UK; ^16^ Academic Department of Medical Genetics University of Cambridge Cambridge UK; ^17^ Northern Genetics Service Newcastle upon Tyne Hospitals Newcastle UK; ^18^ Manchester Centre for Genomic Medicine Central Manchester University Hospitals NHS Foundation Trust Manchester UK; ^19^ Huntsman Cancer Institute University of Utah Health Salt Lake City UT USA; ^20^ Division of Psychosocial Research and Epidemiology Netherlands Cancer Institute Amsterdam The Netherlands

**Keywords:** #pcsm, #ProstateCancer, *BRCA1*, *BRCA2*, psychosocial, quality of life

## Abstract

**Objectives:**

To report the baseline results of a longitudinal psychosocial study that forms part of the IMPACT study, a multi‐national investigation of targeted prostate cancer (PCa) screening among men with a known pathogenic germline mutation in the *BRCA1* or *BRCA2* genes.

**Particpants and Methods:**

Men enrolled in the IMPACT study were invited to complete a questionnaire at collaborating sites prior to each annual screening visit. The questionnaire included sociodemographic characteristics and the following measures: the Hospital Anxiety and Depression Scale (HADS), Impact of Event Scale (IES), 36‐item short‐form health survey (SF‐36), Memorial Anxiety Scale for Prostate Cancer, Cancer Worry Scale‐Revised, risk perception and knowledge. The results of the baseline questionnaire are presented.

**Results:**

A total of 432 men completed questionnaires: 98 and 160 had mutations in *BRCA1* and *BRCA2* genes, respectively, and 174 were controls (familial mutation negative). Participants’ perception of PCa risk was influenced by genetic status. Knowledge levels were high and unrelated to genetic status. Mean scores for the HADS and SF‐36 were within reported general population norms and mean IES scores were within normal range. IES mean intrusion and avoidance scores were significantly higher in *BRCA1*/*BRCA2* carriers than in controls and were higher in men with increased PCa risk perception. At the multivariate level, risk perception contributed more significantly to variance in IES scores than genetic status.

**Conclusion:**

This is the first study to report the psychosocial profile of men with *BRCA1*/*BRCA2* mutations undergoing PCa screening. No clinically concerning levels of general or cancer‐specific distress or poor quality of life were detected in the cohort as a whole. A small subset of participants reported higher levels of distress, suggesting the need for healthcare professionals offering PCa screening to identify these risk factors and offer additional information and support to men seeking PCa screening.

## Introduction

Prostate cancer (PCa) is the most common non‐melanoma tumour in men worldwide, with an estimated 1.1 million men diagnosed with PCa in 2012 1. Men with germline *BRCA1* or *BRCA2* gene mutations are known to be at an increased risk of PCa. This risk is estimated to be 1.8–3.75‐fold and 2.5–8.6‐fold increased by the age of 65 years for *BRCA1* and *BRCA2* mutation carriers, respectively 2, 3. Whilst there is some debate about whether there is a true increased risk of PCa for *BRCA1* mutation carriers, there is solid evidence that *BRCA2* mutation carriers present at a younger age and with aggressive disease 4, 5; therefore, prostate screening and early detection could have an important role in reducing the disease burden, particularly among *BRCA2* mutation carriers 6.

There is controversy about PCa screening using PSA testing in the general population and the benefits and harms of screening have been widely debated 7. The US Prevention Services Task Force currently recommends shared decision‐making for screening healthy men aged 55–69 years 7, 8. Additionally, PCa treatments have significant long‐term side effects that can have an impact on masculine identity, physical and psychosocial symptoms and health‐related quality of life (HRQoL). Research is therefore needed to identify targeted screening tools that can improve the benefit to harm ratio for PCa screening.

The limited number of studies evaluating men with a family history of PCa have generally supported the use of screening in this population 9, 10, 11, 12. To our knowledge, no studies, to date, have prospectively evaluated a PCa screening programme for *BRCA1/2* mutation carriers. The IMPACT study (Identification of Men with a genetic predisposition to ProstAte Cancer: Targeted Screening in men at higher genetic risk and controls) is an international, multicentre study evaluating the role of targeted PSA screening in men with *BRCA1/2* mutations 6.

Evidence supports the theory that genetic testing for *BRCA1* and *BRCA2* mutations does not have a significant long‐term psychological impact on most people tested 13, 14. Studies in men undergoing PCa screening suggest that a minority experience some anxiety, usually while waiting for results 15, 16, 17. Risk factors for anxiety include having a family history of PCa, symptoms or abnormal genetic test results 15, 16, 17. As *BRCA1*/*BRCA2* mutations confer an increased disease risk and psychological distress 18, it is possible that higher levels of anxiety may exist in people with this mutation; however, risk perception has been shown not to reflect true risk in both men with and without a family history of PCa. It has also been reported that cancer worry is high in men with a family history of PCa, with the number of relatives dying from the disease predicting level of worry 18; however, a low level of PCa worry has also been reported in men with a close relative with PCa 19.

Many issues arise when counselling men with *BRCA1*/*BRCA2* mutations, and many factors affect the way in which men react to and use information about their genetic status and risk of developing cancer 20, 21, 22. So far, there have been few investigations either into the HRQoL impact for a man with a *BRCA1*/*BRCA2* mutation living with an increased risk of PCa, or in those men who have gone on to develop PCa 23. Several studies have confirmed the feasibility of collecting HRQoL and psychosocial data as part of large PCa screening trials 16, 24, 25, 26, 27, 28.

In the present study, we report the baseline results of a longitudinal HRQoL investigation carried out as part of the IMPACT study. The specific aims of the study were to evaluate the baseline psychosocial profile of men in the IMPACT study and to identify possible predictors of high levels of psychological distress or poor HRQoL.

## Participants and Methods

### Study Sample and Procedures

The IMPACT study recruited men from families with *BRCA1* or *BRCA2* mutations, with or without the familial mutation, to a programme of annual PCa screening via PSA testing, for a minimum of 5 years. The IMPACT study opened in 2005 and screening will end in 2019. The full design and methods of the IMPACT study have previously been reported 6. The IMPACT study protocol was approved by the West Midlands Research and Ethics Committee in the UK (reference 05/MRE07/25) and subsequently by each participating institution's local ethics committee.

All men eligible for IMPACT were also eligible for the HRQoL study. Men were eligible for participation if they tested either positive, negative or were at 50% risk of inheriting the familial *BRCA1*/*BRCA2* mutation and were aged 40–69 years. Men who tested negative for their familial mutation constituted the control group. Men were excluded if they were known to have PCa at enrolment or if they had another cancer with a prognosis of <5 years survival.

The HRQoL study was added to the IMPACT study protocol in 2009. All sites were invited to participate in this sub‐study. Men enrolled in the IMPACT study at participating sites were approached by letter prior to their next scheduled study appointment inviting them to take part in the HRQoL study. The HRQoL study involves completing a set of questionnaires annually for 5 years, with each assessment taking place prior to the annual PSA test. Men were sent the questionnaires ~4 weeks before their appointment and asked to post it back or bring the completed questionnaire to their appointment. Men were split into two cohorts: (i) a prospective arm, which included men who joined the HRQoL study before their first PSA screening within the IMPACT study; and (ii) a truncated prospective arm, which included men already enrolled in the IMPACT study before joining the HRQoL study. The total target sample was a minimum of 300 men in each arm. In the present analysis, we report the results of the baseline questionnaires in the prospective (not truncated) cohort.

### Study Measures

#### Psychological Distress

Distress was assessed using the Hospital Anxiety and Depression Scale (HADS), the Impact of Event Scale (IES), the Cancer Worry Scale‐Revised (CWS‐R), and the Memorial Anxiety Scale for Prostate Cancer (MAX‐PC). The HADS contains two sub‐scales of seven items that measure the presence and severity of general anxiety and depression 29. Each subscale generates a score ranging from 0 to 21, and a score of >10 indicates clinically relevant levels of anxiety or depression.

The IES is a 15‐item scale measuring PCa‐specific distress through the frequency of intrusive or avoidant thoughts about PCa 30. Total scores on the intrusion and avoidance scales range from 0–35 to 0–40, respectively. A higher score indicates more frequent intrusive/avoidant thoughts about risk of cancer; a score of >8.5 indicates clinically relevant levels of distress.

The CWS‐R is a six‐item scale that measures worry about the risk of developing cancer and the frequency and impact of that worry on mood and daily functioning 31, 32. The CWS‐R uses a score of 1 (no worry) to 4 (maximum worry), giving a summative score between 4 and 24. A high score indicates greater worry, but no clinical thresholds for the scores are available.

The MAX‐PC includes three scales assessing PCa anxiety, PSA anxiety, and fear of recurrence. In the present study, we used the PCa anxiety (11 items) and PSA anxiety (3 items) scales 33. The PCa anxiety scale is scored from 0 to 33 and the PSA anxiety scale from 0 to 9, with a higher score indicating higher anxiety levels.

#### Health‐Related Quality of Life

We assessed HRQoL using the 36‐item short‐form health survey (SF‐36) version 2.0 34, 35. This questionnaire consists of eight subscales: physical functioning; social functioning; role limitations attributable to physical problems; role limitations attributable to emotional problems; mental health; vitality; pain; and general health. Summary scores are calculated for two broad areas of subjective well‐being: physical health and mental health. All scales are linearly converted to a 0–100 scale, with a higher score representing better functioning.

#### Risk Perception

Men were asked to rate their perceived risk of PCa compared with the average man's risk: lower; the same; slightly increased; moderately increased; or strongly increased 36.

#### Knowledge

**Figure 1:**
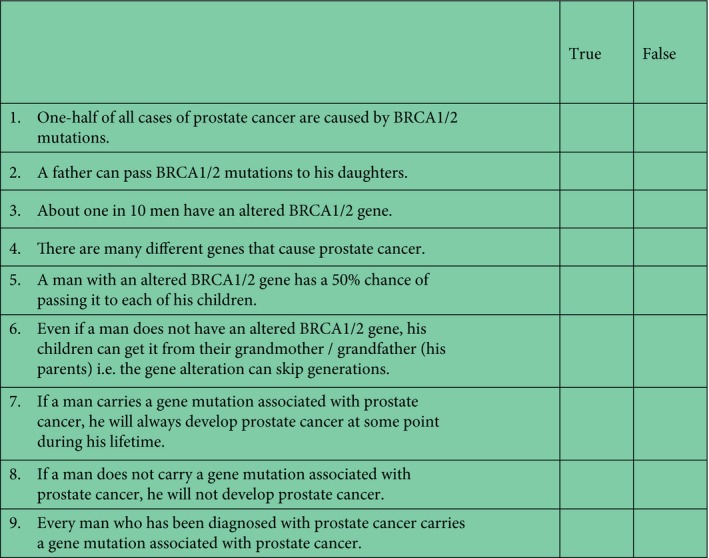
Knowledge questionnaire.

We developed a ‘knowledge’ questionnaire based on a measure developed by Lerman et al. 37 and Wonderlick and Fine 38. The nine true/false items (Fig. 1) assessed knowledge of inheritance of *BRCA1*/*BRCA2*, the effect of having an altered gene, and risk of PCa. Knowledge scores were created by taking the sum of the correct responses to the nine items.

The internal consistency reliability, as assessed by Cronbach's coefficient α, was high for all measures used, ranging from 0.79 for the SF‐36 General Health scale to 0.96 for the SF‐36 Role Physical scale. Fourteen of the 15 scales had an α coefficient >0.80.

### Statistical Analysis

The dataset contained a small amount of missing data. For all scales, except the SF‐36, if ≥75% of a subscale was complete then a total score (corrected for the total number of questions) was calculated. If <75% was completed, data were excluded. For the SF‐36 score, scales were excluded when there was <50% of a sub‐scale completed, as per the recommendation of the scale's authors 39. Ten percent of the data entered were double‐checked for coding accuracy and completeness, and no errors were identified.

The spss 22.0 statistical computer package (SPSS Inc., Chicago, IL, USA) was used to manage and analyse the data. Scores for each questionnaire were calculated in accordance with each scale's scoring system. Descriptive statistics, including means and standard deviations, were used to summarize the sample characteristics and questionnaire data.

All psychometric scales (HADS, IES, SF‐36, MAX‐PC and CWS‐R) were skewed towards better scores. Neither log nor square‐root transformations of these scales produced normal distributions, but given the large sample size within each genetic cohort, parametric tests were used. To minimize the potential effect of multiple testing on the Type I error rate, a *P* value of <0.01 was regarded as statistically significant.

Univariate analysis was used to examine if there were any measurable differences at baseline between *BRCA1* mutation carriers, *BRCA2* mutation carriers and controls on the dependent variables risk perception, HRQoL (SF‐36), the psychological measures (HADS, IES, MAX‐PC) or knowledge. As UK participants made up the largest proportion of participants, a UK dataset was used as a normative comparator for HRQoL, by randomly selecting individuals matched to our sample on age. Means were then compared using a paired Student's *t*‐test 34. Only those aged up to 64 years were recruited to this large population‐based study therefore we limited the analysis to men aged 40–64 years from the IMPACT cohort for the comparison.

The impact of other variables on psychosocial outcomes was also explored. Independent variables included demographics (age, employment status and education), prior PSA screening, family history of PCa, time since genetic testing, and comorbidities coded from clinical interview into a Charlson Comorbidity Index score 40. Knowledge of genetics and PCa and risk perception were also included as independent variables, to examine their impact on psychosocial outcomes.

The associations were investigated initially with anova, Student's *t*‐tests, chi‐squared tests and Pearson's correlations, as appropriate. For categorical independent variables, strength of association was calculated with Cohen's *d* for any significant relationship. Subsequently, multivariate linear regression analyses were performed employing all independent variables found to be associated significantly at the univariate level with a psychosocial outcome.

## Results

### Sample Characteristics and Response Rate

Of the 65 centres participating in the IMPACT study, 23 agreed to take part in the HRQoL sub‐study, including all 19 UK centres, two in Spain and two in the USA. The main reasons for electing not to participate as a centre were financial; there was no specific funding to support this sub‐study at collaborating sites outside of the UK. A total of 780 men enrolled in the HRQoL study, of whom 476 enrolled prior to their first screening visit (prospective cohort, reported in the present paper). This corresponds to 26% of the participants in the IMPACT study taking part in this sub‐study. Those who returned their questionnaire >1 month after their initial screening visit or had not returned the study consent form were excluded (*n* = 35), as were nine men who were untested for their familial mutation, remaining at 50% risk. The data presented are therefore from 432 men, 351 of whom were recruited in the UK, 50 from the USA and 31 from Spain. No significant differences in responses were observed between nationalities.

Uptake into the HRQoL sub‐study was 85–100% at participating sites. There was no significant difference in the participants’ sociodemographics (employment status or education) between the men in this sub‐study and those in the parent IMPACT study.

In all, 98 men (22.7%) carried a mutation in the *BRCA1* gene, 160 (37.0%) carried a mutation in the *BRCA2* gene and 174 (40.3%) were controls. The median time from undergoing genetic testing to joining the IMPACT study was 7.2 months (range 0 months–15.4 years); 47.4% of men joined within 6 months of testing, and 39.6% of men had had at least one PSA measurement before they joined the IMPACT study.

The sociodemographic characteristics and family cancer history of the cohort are shown in Table 1. The mean age of the men when they completed the baseline questionnaire was 53.1 years. The majority were white (98.9%) and in higher managerial or professional occupations (55.3%), and employment and educations levels were similar to the UK general population, with 4.4% unemployed and 37.7% having college degrees or postgraduate qualifications 41, 42.

**Table 1:** Sociodemographic characteristics of the cohort

	*n*	%
Education	415	96.1
Pre‐high school	108	25.0
High school or technical	144	33.3
Degree or postgraduate	163	37.7
Employment	429	99.3
In active paid work	328	75.9
Retired	82	19.0
Unemployed	19	4.4
Family history of prostate cancer	432	100
None	293	67.8
In ≥1 first degree relative	139	32.2
Time since genetic testing	424	98.1
0–3 months prior to enrolment	125	28.9
3–6 months	76	17.6
6–12 months	48	11.1
12–24 months	49	11.3
2–5 years	76	17.6
>5 years	50	11.6
Age	Mean: 53.1; Median: 53.0	SD: 8.5

### Risk Perception and Knowledge

Participants’ perception of their lifetime risk of PCa was influenced significantly by their carrier status (*P* < 0.001; Table 2). *BRCA2* mutation carriers were more likely to rate their risk of PCa as moderately or strongly increased compared with the general population than the control group.

**Table 2:** Descriptive statistics and summary of group comparisons for the psychosocial variables

Scale	Scale range / threshold		Overall		*BRCA1* mutation carriers		*BRCA2* mutation carriers		Controls		Cohen's *d* *
			*N*	Mean (sd) % above threshold	*N*	Mean (sd) % above threshold	*N*	Mean (sd) % above threshold	*N*	Mean (sd) % above threshold	
SF‐36 physical component summary	Range	0–100	404	47.4 (10.0)	90	46.4 (10.7)	148	47.1 (10.1)	166	48.3 (8.6)	
SF‐36 mental component summary	Range	0–100	404	52.4 (10.2)	90	52.1 (11.1)	148	51.2 (10.5)	166	53.7 (9.3)	
Total anxiety (HADS)	Range	0–21	431	4.9 (3.6)	97	4.9 (3.5)	160	4.8 (3.8)	174	4.9 (3.4)	
	Abnormal threshold	≥11	28	6.5%	6	6.2%	12	7.5%	10	5.7%	
Total depression (HADS)	Range	0–21	431	2.8 (3.0)	97	2.9 (3.2)	160	2.9 (3.1)	174	2.7 (2.7)	
	Abnormal threshold	≥11	9	2.1%	3	3.1%	4	2.5%	2	1.1%	
Total intrusion (IES)	Range	0–35	423	2.3 (4.9)	94	**3.0** † (5.7)	158	**3.1** † (5.5)	171	**1.3** † (3.5)	−0.02; 0.35; 0.38
	Abnormal threshold	≥19	12	2.8%	4	4.3%	6	3.8%	2	1.2%	
Total avoidance (IES)	Range	0–40	418	4.3 (7.0)	93	**6.0** † (8.4)	156	**5.1** † (7.4)	169	**2.6** † (5.2)	0.11; 0.48; 0.39
	Abnormal threshold	≥19	32	7.7%	12	12.9%	15	9.6%	5	3.0%	
Total MAX‐PC	Range	0‐33	420	3.5 (5.4)	94	4.1 (5.5)	156	3.9 (6.2)	170	2.8 (4.6)	
Total cancer worry	Range	4–24	430	9.5 (2.5)	97	**9.7** † (2.7)	160	**9.9** † (2.7)	173	**9.1** † (2.0)	−0.09; 0.25; 0.36
Risk perception			423	N/A	91	N/A	156	N/A	171	N/A	
	Moderately or strongly increased		133	31.4%	31	**32.3%** ‡	86	**55.1%** ‡	16	**9.4%** ‡	0.43§
Total knowledge score	Range	0–9	404	7.1 (1.7)	92	6.9 (1.8)	151	7.2 (1.6)	161	7.1 (1.7)	

HADS, Hospital Anxiety and Depression Scale; IES, Impact of Event Scale; MAX‐PC, Memorial Anxiety Scale for Prostate Cancer; SF‐36, 36‐item short‐form health survey. *Cohen's *d* values are listed comparing *BRCA1* mutation carriers with *BRCA2* mutation carriers; *BRCA1* mutation carriers with controls; *BRCA2* mutation carriers with controls. ^†^ *P* < 0.01 using ANOVA. ^†^ *P* < 0.01 using a chi‐squared test for independence. ^§^Cramer's V test for nominal association. Bold font indicates statistically significant values.

Knowledge scores were not affected by the genetic status of the participant, time since genetic testing or education level. Family history of PCa, education level, time since genetic testing and age were not significantly associated with any of the outcome variables.

### SF‐36 Questionnaire

Overall physical functioning SF‐36 scores did not differ significantly from the normative sample (IMPACT sample aged 40–64 years mean score: 48.1; matched normative sample mean score: 47.5; *P* = 0.52). The overall mental functioning SF‐36 score was significantly better in the study cohort compared with the normative sample, but the effect size was small and both mean values were close to the standardized mean of 50 (IMPACT sample aged 40–64 years mean score: 52.0; matched normative sample mean score: 49.8; *P* = 0.008, Cohen's *d* = 0.21). Means also did not differ significantly across genetic groups.

### HADS Questionnaire

The overall mean anxiety and depression scores for the HADS were 4.9 and 2.8, respectively, which were not higher than previously reported general population norms 43. The means across different genetic risk groups also did not differ significantly (Table 2; anxiety: *P* = 0.99; depression: *P* = 0.75).

None of the independent variables showed a significant association with either the anxiety or depression scores. Those with higher risk perception had slightly higher scores on the anxiety and depression scales (*P* = 0.02 and *P* = 0.03, respectively; Table 3), although the difference was not clinically significant.

**Table 3:** Means of psychosocial scales according to risk perception categories

Scale (mean scores)	Risk perception		*P*	Cohen's *d*
	Not or slightly increased	Moderately/strongly increased		
HADS anxiety	4.54	5.43	0.02	
HADS depression	2.55	3.23	0.03	
IES intrusion	1.33	4.42	<0.001	−0.57
IES avoidance	3.32	6.11	0.001	−0.39
MAX‐PC (PCa)	2.62	5.32	<0.001	−0.47
CWS‐R	8.89	10.84	<0.001	−0.76

CWS‐R, Cancer Worry Scale‐Revised; HADS, Hospital Anxiety and Depression Scale; IES, Impact of Event Scale; MAX‐PC, Memorial Anxiety Scale for Prostate Cancer; PCa, prostate cancer; SF‐36, 36‐item short‐form health survey.

### IES, CWS, MAX‐PC Questionnaires

At the univariate level, the mean intrusion and avoidance scores on the IES scale were significantly higher in both *BRCA1* and *BRCA2* mutation carriers compared with controls (intrusion: *P* = 0.001; avoidance: *P* < 0.001; Table 2) and higher in those who perceived their PCa risk as moderately or strongly increased (intrusion: *P* < 0.001, avoidance: *P* = 0.001; Table 3); however, at the multivariate level, risk perception contributed more significantly to the variation in IES scores than genetic status (Table 4).

**Table 4:** Results of multivariable linear regression analysis for the Hospital Anxiety and Depression Scale, Impact of Events Intrusion and Avoidance, and Cancer Worry Scale‐Revised

	Variables	*B*	se	*T*	*P*	*R* ^2^	*R* ^2^ change
IES intrusion	Risk perception	2.92	0.55	5.32	<0.001	0.087	0.087
	*BRCA2* status	0.42	0.58	0.72	0.47	0.087	0.000
	*BRCA1* status	0.98	0.62	1.59	0.11	0.092	0.006
IES avoidance	Risk perception	2.18	0.81	2.70	0.007	0.058	0.017
	*BRCA2* status	1.50	0.85	1.76	0.08	0.042	0.025
	*BRCA1* status	2.88	0.91	3.18	0.002	0.017	0.017
CWS‐R	Risk perception	1.98	0.27	7.46	<0.001	0.137	0.137
	*BRCA2* status	–0.07	0.28	–0.24	0.81	0.138	0.001
	*BRCA1* status	0.14	0.30	0.47	0.64	0.138	0.000

CWS‐R, Cancer Worry Scale‐Revised; HADS, Hospital Anxiety and Depression Scale; IES, Impact of Event Scale. Variables included represent those significant on the univariate level.

A similar pattern was seen for the cancer worry score. Scores were generally low and univariately associated with genetic status (CWS‐R: *P* = 0.004; Table 2) and risk perception (CWS: *P* < 0.001; Table 3). Again, risk perception was more highly associated with higher cancer worry than genetic status in the multivariate model (Table 4).

Scores for PCa anxiety (MAX‐PC) were only associated with risk perception (*P* < 0.001), therefore, a multivariate analysis was not undertaken.

## Discussion

This study investigated the baseline HRQoL and psychosocial profiles of men taking part in the IMPACT study, prior to their first screening appointment. The results indicate that participants, in general, do not have clinically concerning levels of general or cancer‐specific distress (i.e. indicative of the presence of clinical depression or anxiety) or poor HRQoL. A small subset of participants had higher levels of distress, but perception of risk contributed more to explaining the variance in distress level than did genetic status. General population screening studies in the UK and European series have reported similar findings: that PCa screening does not have a detrimental effect on measures of HRQoL and psychological health 28, 44, 45.

It was reassuring that participants’ perceptions of PCa risk were influenced by carrier status, largely reflecting what would have been communicated during genetic counselling 2, 3. As expected, *BRCA2* mutation carriers had the highest perceived risk of PCa, most frequently classifying risk as ‘slightly’ or ‘moderately’ increased, and controls most frequently classifying risk as the ‘same’ as the general population.

Knowledge levels were high across the cohorts, irrespective of genetic status, education level and time since testing, showing that men retained accurate information about inheritance of *BRCA1*/*BRCA2* mutations and cancer risk. The knowledge questionnaire was designed specifically for this study, but was adapted from that used in other studies 37, 38. These studies reported knowledge levels to be ~50% in women at risk of breast cancer prior to *BRCA1*/*BRCA2* testing. The high levels of knowledge reported in our cohort could reflect that they have recently revisited their risk status in making a decision to undergo screening in the IMPACT study; however, men were asked to complete these questionnaires prior to their first screening appointment and so may not have had a detailed discussion about risk of PCa since being informed about their genetic status.

The sociodemographic characteristics of the cohort indicate that employment and education levels are similar to those observed in the UK general population 41, 42; however, participants were predominantly white, which is not representative of the general UK population, and therefore caution should be exercised in generalizing these results to other ethnic groups.

The HRQoL assessments did not detect any clinically relevant differences in either physical or mental health when compared with general population samples, both matched and unmatched by age 34. Our results support those of the Finnish European Randomized Screening for Prostate Cancer study cohort in which HRQoL was also assessed with the SF‐36 45. As in our cohort, HRQoL scores were observed to be higher than in the general Finnish population 45, but not at clinically significant levels; this was hypothesized to be because the men were generally healthy and well educated; however the Finnish cohort was not age‐matched, which may have conferred some bias.

In terms of general distress, scores were within previously reported population norms 43 and no differences were observed between mutation carriers and controls. For cancer‐specific distress, a significant difference was found between *BRCA* mutation carriers and controls for both the IES and CWS; however the differences were small and mean scores remained below clinically relevant levels for the IES. Importantly, at the multivariate level, risk perception was found to have a stronger association with distress levels than genetic status itself.

No significant association was observed between anxiety and having a family history of PCa, supporting previous reports 15, 24, 28, 44, 46. Men reporting higher PCa risk perception were found to have consistently higher scores across all psychological distress scales (general and cancer‐specific). Similar results were reported by Taylor et al. 24; however, the effect size was small across all scales and no group had a mean distress score that reached clinically significant levels, where such thresholds were available 30, 43. It is therefore fair to conclude that, whilst having a modest impact on men's distress levels, a high perceived PCa risk is not associated strongly with clinically significant levels of distress in this cohort.

A number of studies have reported that anxiety about cancer screening affects a small number of people who are predisposed to anxiety, and that this anxiety continues throughout participation in cancer screening 16, 27, 28, 44, 47. Our data support this finding, with a small proportion of men reporting clinically significant levels of distress. It will be important to compare these baseline levels with subsequent screening rounds in the IMPACT study and to include previous high PSA results as a covariate, as both the European and US screening studies report high levels of anxiety in men with previously elevated PSA levels 26, 27. Identifying men with a predisposition to high levels of psychological distress could facilitate providing timely support to manage this distress and potentially increase adherence to screening recommendations.

We did not observe a significant association between distress and age. While this supports several earlier studies 16, 44, one study reported an inverse relationship between age and distress levels 27.

It is important to consider whether we would have observed different results if all men in the IMPACT study had been included in this psychosocial sub‐study; however we found no difference in sociodemographic characteristics between the men in the sub‐study and those in the IMPACT study as a whole. It could be that those more predisposed to anxiety may be inclined not to join the psychosocial sub‐study; however, no evidence of this has been found by others 28.

We obtained a very high uptake level for the psychosocial sub‐study, with at least 85% opting in at participating sites. Uptake was also found to be high in the European Randomized study of Screening for Prostate Cancer Swedish cohort, with 84–94% of men with abnormal PSA levels completing a questionnaire measuring anxiety levels 27. The high participation rate is probably attributable to the embedding of this psychosocial study into an existing screening study, and therefore inviting participants who are already highly motivated to contribute to research.

A strength of the present study is the use of a number of different, standardized psychological measures that offer extensive insight into the psychosocial profile of the participants and that allow comparison of the results with a number of other PCa screening studies that have used the same or similar measures.

It should be noted that our sample was restricted to men who have previously engaged with health services by undergoing genetic testing and who responded positively to an invitation to take part in a research study. In addition, there was limited variability in ethnicity, which may limit the generalizablity of the findings to other populations.

The data presented represent a snapshot of men's psychosocial profiles when they joined the IMPACT study. Follow‐up data will inform whether the PCa screening process has an impact on HRQoL or distress over time.

To the best of our knowledge, this is the first study to report the psychosocial and HRQoL profile of men with *BRCA1*/*BRCA2* mutations taking part in a PCa screening study. Uptake into the study was very high, and participants had very high levels of knowledge about genetics and PCa. As a whole, the cohort did not demonstrate any clinically concerning levels of general or cancer‐specific distress or poor HRQoL. A small subset of participants reported higher levels of distress, but perception of risk was more strongly associated with distress levels than was genetic status. It is important for healthcare professionals who are providing PCa screening to be aware of these predictors of distress so that men with potential for heightened distress can be identified and adequate counselling and support can be offered. Follow‐up data will determine whether these factors have an impact on adherence to screening and whether men experiencing abnormal PSA results experience more distress.

## Conflict of Interest

Prof. Rosalind Eeles: Janssen Pharmaceutica, provided medical education support to GU ASCO February 2013, and received an honorarium and expenses for attending and speaking at the UK Cancer Convention, October 2013 from Succinct Communications. The remaining authors have no other conflict of interest to declare.

**Abbreviations**
CWS‐R: Cancer Worry Scale‐Revised
HADS: Hospital Anxiety and Depression Scale
HRQoL: health‐related quality of life
IES: Impact of Event Scale
MAX‐PC: Memorial Anxiety Scale for Prostate Cancer
PCa: prostate cancer
SF‐36: 36‐item short‐form health survey

## Supporting information


**Appendix S1.** The IMPACT Collaborators.
