# Supplementary material for: Psychosocial impact of undergoing prostate cancer screening for men with BRCA1 or BRCA2 mutations
Source: BJU Int. 2018 Jun 22;123(2):284–92. doi: 10.1111/bju.14412 (PMC6378691; doi:10.1111/bju.14412)
Supplement: Supplementary file 1 — Appendix S1. The IMPACT Collaborators. [file BJU-123-284-s001.doc]

**Appendix 1 - The IMPACT Collaborators**

**IMPACT Study Steering Committee:**

**PI: Professor Rosalind Eeles –** Institute of Cancer Research, London, UK

**Ms Elizabeth Bancroft -** Royal Marsden NHS Foundation Trust, London, UK

**Ms Elizabeth Page -** Institute of Cancer Research, London, UK

**Dr Zsofia Kote-Jarai -** Institute of Cancer Research, London, UK

**Mrs Audrey Ardern-Jones –** Royal Marsden NHS Foundation Trust, London, UK

**Prof Dr Chris Bangma -** Erasmus University Medical Center, Rotterdam, The Netherlands

**Dr Elena Castro –** CNIO, Madrid, Spain

**Professor David Dearnaley -** Institute of Cancer Research, London, UK

**Dr Alison Falconer –** Imperial College Healthcare NHS Trust, London, UK

**Professor Christopher Foster -** HCA Pathology Laboratories, London, UK

**Professor Henrik Grönberg -** University Hospital, Umea, Sweden

**Professor Freddie C. Hamdy -** University of Oxford, Oxford, UK

**Dr Óskar Þór Jóhannsson -** Landspitali - National University Hospital of Iceland, Reykjavik, Iceland

**Dr Vincent Khoo -** Royal Marsden NHS Foundation Trust, London, UK

**Professor Diana Eccles** – Wessex Clinical Genetics Service, Southampton, UK

**Professor Hans Lilja –** MSKCC, New York & University of Oxford, Oxford, UK

**Professor Gareth Evans -** St Mary’s Hospital, Manchester, UK

**Professor Jorunn Eyfjord -** University of Iceland, Reykjavik, Iceland

**Professor Jan Lubinski -** International Hereditary Cancer Center, Szczecin, Poland

**Dr Lovise Maehle -** Norwegian Radium Hospital, Oslo, Norway

**Dr Christos Mikropoulos** – The Institute of Cancer Research, London, UK

**Mr Alan Millner -** Royal Marsden NHS Foundation Trust, London, UK

**Dr Anita Mitra –** University College London Hospitals, London, UK

**Dr Judith Offman -**  **Dr Judith Offman -**  King’s College London, UK

**Ms Clare Moynihan -** Institute of Cancer Research, London, UK

**Dr Gad Rennert -** CHS National Cancer Control Center, Carmel Medical Center, Haifa, Israel

**Dr Mohnish Suri -** Nottingham City Hospital, Nottingham, UK

**Coordinating Centre, Institute of Cancer Research, London:** Rosalind Eeles, Elizabeth Bancroft, Elizabeth Page, Sibel Saya, Alex Dias, Natalie Taylor, Kathryn Myhill, Sarah Thomas, Lucia D’Mello, Jenny Pope, Anthony Chamberlain

**Australia (*more than 1 affiliation)**

**Parkville Familial Cancer Centre, Peter MacCallum Cancer Centre, East Melbourne, VIC:** Paul James*, Gillian Mitchell*, Sue Shanley, Kate Richardson, Joanne McKinley, Lara Petelin, Morgan Murphy, Lyon Mascarenhas

**The Sir Peter MacCallum Department of Oncology, University of Melbourne, VIC**: Gillian Mitchell*, Paul James*

**Genetics Medicine, Royal Melbourne Hospital, Melbourne, VIC:** Paul James*

**Department of Urology, Peter MacCallum Cancer Centre, East Melbourne, VIC**: Declan Murphy

**Department of Urology, Repatriation General Hospital, Daw Park, SA**: Jimmy Lam, Louise Taylor, Cathy Miller, Alan Stapleton, Michael Chong

**SA Clinical Genetics Service, SA Pathology (at Women's & Children's Hospital), North Adelaide, SA**: Graeme Suthers, Nicola Poplawski

**Hereditary Cancer Clinic, Prince of Wales Hospital, Randwick, NSW**: Katherine Tucker*, Lesley Andrews, Jessica Duffy

**Department of Urology, Prince of Wales Hospital, Randwick, NSW**: Richard Millard

**Hereditary Cancer Clinic, Prince of Wales Clinical School, Faculty of Medicine, UNSW, Sydney, NSW:** Robyn Ward, Rachel Williams

**St Vincent's Clinic, Sydney, NSW:** Phillip Stricker

**Familial Cancer Service, Westmead Hospital, Wentworthville, NSW**: Judy Kirk*, Michelle Bowman

**Centre for Cancer Research, The Westmead Institute for Medical Research, NSW**: Judy Kirk*

**Westmead Hospital, Wentworthville, NSW**: Manish Patel

**Familial Cancer Centre, Monash Health, Clayton, VIC**: Marion Harris, Shona O'Connell, Clare Hunt, Courtney Smyth

**Monash Medical Centre, VIC**: Mark Frydenberg

**Parkville Familial Cancer Centre, The Royal Melbourne Hospital, Grattan St, Parkville, VIC**: Geoffrey Lindeman*, Kylie Shackleton, Catherine Morton

**Stem Cells and Cancer Division, The Walter and Eliza Hall Institute of Medical Research, Parkville, VIC:** Geoffrey Lindeman*

**Department of Medicine, The University of Melbourne, Parkville, VIC**: Geoffrey Lindeman*

**Genetic Health Queensland, Royal Brisbane & Women's Hospital, Herston, QLD**: Rachel Susman, Julie McGaughran, Melanie Boon

**Genetic Services of WA, King Edward Memorial Hospital, Subiaco, WA**: Nicholas Pachter*, Sharron Townshend, Lyn Schofield, Cassandra Nicholls

**Dept of Paediatrics, University of Western Australia, Perth, WA**: Nicholas Pachter*

**Hunter Family Cancer Service, Waratah, NSW**: Allan Spigelman*, Margaret Gleeson

**University of New South Wales, St Vincent’s Clinical School, NSW**: Allan Spigelman*

**Hereditary Cancer Clinic ,** **The Kinghorn Cancer Centre, St Vincent's Hospital, Sydney, NSW**: Allan Spigelman*

**Tasmanian Clinical Genetics Service, Hobart, TAS**: David Amor*, Jo Burke, Briony Patterson

**Murdoch Childrens Research Institute, Parkville, VIC**: David Amor*

**Mater Private Hospitals, QLD**: Peter Swindle

**School of Biomedical Sciences and Pharmacy, University of Newcastle, NSW**: Rodney Scott

**Victorian Cancer Biobank, Carlton, VIC**

**Department Gynaecological Oncology Laboratory, Westmead Hospital, Centre for Cancer Research, NSW**

**PathWest (Clinical Trials Lab), Nedlands, WA**

**Pathology Queensland (Central Laboratory; Health Support Queensland), QLD Department of Health, Herston, QLD**

**Canada**

**McGill University, Montreal:** William Foulkes, Talia Boshari, Armen Aprikian.

**Denmark**

**Vejle Hospital, Vejle, Denmark:** Thomas Jensen, Anders Bojeson, Palle Osther, Anne-Bine Skytte, Dorthe Cruger, Majbritt Kure Tøndering

**Odense University Hospital, Odense:** Anne-Marie Gerdes

**Germany**

**Center of Familial Breast and Ovarian Cancer, University Hospital of Cologne, Cologne:**

Rita Schmutzler, Kerstin Rhiem, Petra Wihler

**University Hospital Dresden:** Dr K Kast, C Griebsch

**Iceland**

**University Hospital of Iceland, Reykjavik:** Oskar Johannsson, Vigdis Stefansdottir

**India**

**Tata Memorial Centre, Mumbai:** Vedang Murthy, Rajiv Sarin, Kasturi Awatagiri, Sujata Ghonge, Pradnya Kowtal, Gouri Mulgund

**Ireland**

**Mater Private Hospital, Dublin:** David Gallagher, Richard Bambury, Michael Farrell, Fergal Gallagher, Ingrid Kiernan

**Israel**

**Chaim Sheba Medical Center, Tel-Hashomer and the Sackler School of Medicine, Tel-Aviv University, Tel-Aviv:** Eitan Friedman

**The Genetic Institute, the Gastroenterology Institute and the Urology Department, Kaplan Medical Centre, Rehovot:** Rakefet Chen-Shtoyerman, Alon Basevitch, Dan Leibovici, Ehud Melzer and Sagi Josefsberg Ben-Yehoshua

**Italy**

**Istituto Nazionale dei Tumori, Milan:** Nicola Nicolai, Paolo Radice, Riccardo Valdagni, Tiziana Magnani, Simona Gay

**Malaysia**

**Cancer Research Initiatives Foundation, Subang Jaya Medical Centre, Selangor Darul Ehsan:** Soo Hwang Teo, Hui Meng Tan, Sook-Yee Yoon

**University of Malaya, Kuala Lumpur:** Soo Hwang Teo, Meow Keong Thong

**The Netherlands**

**STOET(Stichting Opsporing Erfelijke Tumoren), Leiden**: Hans Vasen, Janneke Ringleberg

**Leiden University Medical Centre, Leiden:** Christi van Asperen.

**Radboud University Nijmegen Medical Centre:** Bart Kiemeney. Wendy van Zelst-Stams

**University Medical Center Utrecht**: Margreet G.E.M. Ausems, Rob B. Van der Luijt

**Academic Medical Center , Amsterdam:** Theo van Os

**Netherlands Cancer Institute, Amsterdam**: Mariëlle W.G. Ruijs

**VU University Medical Center, Amsterdam**: Muriel A. Adank

**Erasmus Medical Center, Rotterdam**: Rogier A. Oldenburg

**University Hospital Maastricht** : A. (Paula) T.J.M. Helderman- van den Enden, B.A.H. Caanen

**University Medical Centre Groningen**: Jan C. Oosterwijk

**Norway**

**Norwegian Radium Hospital, Oslo:** Lovise Maehle, Pal Moller, Bjorn Brennhovd, Heidi Medvik, Eldbjørg Hanslien, Eli Marie Grindedal

**Poland**

**International Hereditary Cancer Centre, Szczecin:** Cezary Cybulski, Jan Lubinski, Dominika Wokolorczyk

**Portugal**

**Portuguese Oncology Institute, Porto:** Manuel Teixeira, Sofia Maia, Ana Peixoto, Rui Henrique, Jorge Oliveira, Nuno Gonçalves, Luís Araújo, Manuela Seixas, João Paulo Souto, Pedro Nogueira

**Slovakia**

**National Cancer Institute, Bratislava:** Lucia Copakova.

**Slovenia**

**Institute of Oncology, Ljubljana:** Janez Zgajnar, Mateja Krajc, Alenka Vrecar.

**Spain**

**Hereditary Cancer Program, Catalonian Institute of Oncology, Barcelona:** Monica Salinas, Gabriel Capellá,

**Hospital de Sant Pau, Barcelona:** Teresa Ramón y Cajal, David Fisas, Josefina Mora, Salvador Esquena

**Hospital Vall d'Hebron, Barcelona:** Judith Balmaña, Neus Gadea, Juan Morote

**Sweden**

**Karolinska University Hospital, Karolinska Institutet, Stockholm:** Annelie Liljegren, Marie Hjälm –Eriksson, Karl-Johan Ekdahl, Stefan Carlsson

**United Kingdom**

**Royal Marsden NHS Foundation Trust:** Angela George, Zoe Kemp, Jennifer Wiggins, Cathryn Moss, Vincent Khoo, Nicholas Van As, Alan Thompson, Chris Ogden, Christopher Woodhouse, Pardeep Kumar.

**Manchester Regional Genetics Service, Manchester:** D Gareth Evans, Barbara Bulman, Jeanette Rothwell, Karen Tricker.

**Wessex Clinical Genetics Service, Southampton:** Diana Eccles, Gillian Wise, Catherine Mercer, Donna McBride, Philandra Costello, Allison Pearce, Audrey Torokwa.

**East Anglian Regional Genetics Service, Cambridge:** Marc Tischkowitz, Joan Paterson, Virginia Clowes, Amy Taylor, Barbara Newcombe.

**Oxford Regional Genetics Service, Oxford:** Lisa Walker, Dorothy Halliday, Barbara Stayner, D Fleming-Brown

**South West Thames Regional Genetics Service, London:** Katie Snape, Helen Hanson, Shirley Hodgson, Glen Brice, Tessa Homfray, Carrie Hammond, Kelly Kohut, Uruj Anjum, Audrey Dearing, Mark Mencias.

**Peninsula Clinical Genetics Service. Exeter:** Carole Brewer, Alison Potter, Caroline Renton, Anne Searle, Kathryn Hill, Selina Goodman, Lynda Garcia, Gemma Devlin, Sarah Everest, Maria Nadolski.

**Northern Clinical Genetics Service, Newcastle:** Alex Henderson, Fiona Douglas, Irene Jobson, Edgar Paez.

**South West Regional Genetics Service, Bristol:** Alan Donaldson, Sue Tomkins.

**South East Thames Regional Genetics Service, Guys Hospital London:** Caroline Langman, Chris Jacobs, Gabriella Pichert, Adam Shaw, Anju Kulkarni, Vishakha Tripathi, Sarah Rose, Cecilia Compton, Michelle Watson and Cherylin Reinholtz.

**North West Thames Regional Genetics Service, Harrow:** Angela Brady, Virginia Clowes, Huw Dorkins, Athalie Melville, Monika Kosicka-Slawinska, Carole Cummings, Vicki Kiesel, Marion Bartlett, Kashmir Randhawa, Natalie Ellery

**North East Thames Regional Genetics Service, NE Thames:** Lucy Side, Alison Male, Kate Simon, Katie Rees, Cecilia Compton, Lizzie Tidey, Jana Gurasashvili.

**North Trent Clinical Genetics Service, Sheffield:** Jackie Cook, Louise Nevitt, Stuart Ingram, Alice Howell

**Academic Urology Unit, Sheffield:** Derek Rosario, James Catto, Joanne Howson.

**West Midlands Regional Clinical Genetics Service, Birmingham:** Rachel Hart, Kai-Ren Ong, Cyril Chapman, Trevor Cole, Tricia Heaton, Jonathan Hoffman, Lucy Burgess, Wayne Glover, Camilla Huber, Farah Islam

**West of Scotland Genetics Service, Glasgow:** Rosemarie Davidson, Mark Longmuir, Cathy Watt, Alexis Duncan

**Leicester Royal Infirmary:** Julian Barwell, Roger Kockelbergh, Shumikazi Mzazi, Amy Dineen, Ayisha Sattar, Beckie Kaemba, Zahirah Sidat, Nafisa Patel, Kas Siguake

**North Cumbria University Hospitals Trust:** Alex Henderson, Angela Birt, Una Poultney, Nkem Umez-Eronini, Jaswant Mom

**Cheshire and Mersey Clinical Genetics Service, Liverpool Women’s Hospital. Lynn Greenhalgh, Vivienne Sutton**

**Royal Liverpool and Broadgreen Hospital NHS Trust, Liverpool:** Philip Cornford, Nicola Bermingham, Pembe Yesildag, Katy Treherne, Julie Griffiths

**Derriford Hospital, Plymouth:** Carole Brewer, Lyn Cogley, Hannah Gott

**United States**

**NorthShore University HealthSystem, Evanston:** Dr. Wendy S Rubinstein, Dr. Peter Hulick, Dr. Michael McGuire, Dr. Daniel Shevrin, Dr. Karen Kaul, Scott Weissman CGC, Anna Newlin , Kristen Vogel , Shelly Weiss, Nicole Hook

**Salt Lake City, Utah:** Saundra Buys, David Goldgar, Tom Conner, Vickie Venne, Robert Stephenson, Christopher Dechet

**University of Pennsylvania, Philadelphia:** Susan Domchek, Jacquelyn Powers, Neil Rustgi

**MD Anderson, Texas:** Sara Strom, Banu Arun, John W. Davis, Yuko Yamamura

**Fox** **Chase Cancer Center:** Elias Obeid, Veda Giri, Laura Gross, Lisa Bealin

**University of Michigan:** Kathy Cooney, Elena Stoffel, Linda Okoth
